# Detaching from the negative by reappraisal: the role of right superior frontal gyrus (BA9/32)

**DOI:** 10.3389/fnbeh.2014.00165

**Published:** 2014-05-09

**Authors:** Rosalux Falquez, Blas Couto, Agustin Ibanez, Martin T. Freitag, Moritz Berger, Elisabeth A. Arens, Simone Lang, Sven Barnow

**Affiliations:** ^1^Department of Clinical Psychology and Psychotherapy, Institute of Psychology, University of HeidelbergHeidelberg, Germany; ^2^Laboratory of Experimental Psychology and Neuroscience, Institute of Cognitive Neurology, Favaloro UniversityBuenos Aires, Argentina; ^3^UDP-INECO Foundation Core on Neuroscience, Diego Portales UniversitySantiago, Chile; ^4^Departamento de Psicología, Universidad Autónoma del CaribeBarranquilla, Colombia; ^5^Department of Radiology, German Cancer Research CenterHeidelberg, Germany

**Keywords:** emotion regulation, self-focused reappraisal, VLSM, VBM, right SFG

## Abstract

The ability to reappraise the emotional impact of events is related to long-term mental health. Self-focused reappraisal (REAPPself), i.e., reducing the personal relevance of the negative events, has been previously associated with neural activity in regions near right medial prefrontal cortex, but rarely investigated among brain-damaged individuals. Thus, we aimed to examine the REAPPself ability of brain-damaged patients and healthy controls considering structural atrophies and gray matter intensities, respectively. Twenty patients with well-defined cortex lesions due to an acquired circumscribed tumor or cyst and 23 healthy controls performed a REAPPself task, in which they had to either observe negative stimuli or decrease emotional responding by REAPPself. Next, they rated the impact of negative arousal and valence. REAPPself ability scores were calculated by subtracting the negative picture ratings after applying REAPPself from the ratings of the observing condition. The scores of the patients were included in a voxel-based lesion-symptom mapping (VLSM) analysis to identify deficit related areas (ROI). Then, a ROI group-wise comparison was performed. Additionally, a whole-brain voxel-based-morphometry (VBM) analysis was run, in which healthy participant's REAPPself ability scores were correlated with gray matter intensities. Results showed that (1) regions in the right superior frontal gyrus (SFG), comprising the right dorsolateral prefrontal cortex (BA9) and the right dorsal anterior cingulate cortex (BA32), were associated with patient's impaired down-regulation of arousal, (2) a lesion in the depicted ROI occasioned significant REAPPself impairments, (3) REAPPself ability of controls was linked with increased gray matter intensities in the ROI regions. Our findings show for the first time that the neural integrity and the structural volume of right SFG regions (BA9/32) might be indispensable for REAPPself. Implications for neurofeedback research are discussed.

## Introduction

Cognitive emotion regulation (ER) is conceptualized as the ability to modulate the spontaneous flow of emotional states by means of cognitive control. That means, the ability to manage not only which emotion we feel, but when and how this emotion is experienced and expressed (Gross, 1999). Thus, people can effectively take control over their own emotional responses by adjusting them to everyday events and context demands. Conversely, disturbances in ER abilities might disrupt human adaptation and therefore compromise well-being as well as lead to unhealthy social functioning (Aldao and Dixon-Gordon, 2013). For instance, previous studies have emphasized that an impaired ER constitutes a core feature of affective (Goldin et al., 2009; Hermann et al., 2009; New et al., 2009; Abler et al., 2010) and personality disorders (Slee et al., 2008; Lang et al., 2012). According to Gross's *process model of ER*, some regulation strategies might be more protective than others, particularly when they act early in the emotion-generative process (Gross, 1999, 2002). The early change of the way an emotional stimulus is appraised in order to decrease its emotional impact (i.e., reappraisal) is associated with long-term psychological health outcomes (Gross and John, 2003; Goldin et al., 2008; Barnow, 2012). Reappraisal is held to be very effective for the down-regulation of negative emotions, as it has been shown to decrease peripheral psychophysiology (Ray et al., 2010; Kim and Hamann, 2012) and self-reported negative affect (Gross, 1998; Ochsner et al., 2002). Moreover, previous studies investigating neural activation pattern during reappraisal show decreased activation of emotional limbic regions such as the amygdala (Ochsner et al., 2002; Banks et al., 2007; Wager et al., 2008).

Importantly, the down-regulation of emotional limbic regions through reappraisal has been shown to occur by means of top-down influences of cognitive control regions in the prefrontal cortex (PFC; Buhle et al., 2013). Reappraisal is one of the most complex strategies; it involves a variety of cognitive control abilities which serve to generate alternative explanations about an emotionally arousing cue. For example, participants need to rely on working memory (WM) so as to keep or update alternative reinterpretations in mind (Hofmann et al., 2012; Schweizer et al., 2013). Furthermore, flexibility skills are crucial in order to choose between new reinterpretations (Joormann and Gotlib, 2008; Malooly et al., 2013). Also, cognitive inhibition skills are similarly important, especially for the decrease of automatic emotional appraisals (Joormann, 2010; Pe et al., 2013). Following, all of these processes need to be monitored in order to keep track of the regulation success according to internal and external demands (Paret et al., 2011). In line with these assumptions, neural structures commonly shown in functional magnetic resonance imaging (fMRI) investigations examining reappraisal of negative stimuli include mainly regions in the superior frontal gyrus (SFG), like the dorsolateral PFC (dlPFC; BA9/46) and the anterior cingulate cortex (ACC, BA32; Harenski and Hamann, 2006; Banks et al., 2007; Erk et al., 2010; Leiberg et al., 2012; Ochsner et al., 2012). These regions are found to be highly involved in WM and inhibition performance (Lutcke and Frahm, 2008; Shackman et al., 2009; Balconi, 2013). Similarly, an increased blood-oxygen-level-dependent (BOLD) contrast of the dorsal ACC (dACC;BA32) has been observed during reappraisal of negative social situations (Koenigsberg et al., 2010), as well as during situations where error monitoring (van Veen et al., 2001; Ichikawa et al., 2011) and cognitive flexibility are required (Zastrow et al., 2009; Vriend et al., 2013). Therefore, reappraisal function relies to a great extent on the same regions involved in several complex cognitive control tasks (Schweizer et al., 2013).

Besides its functional complexity, there are several types of reappraisal (McRae et al., 2012a). To date, two main variants of strategies have been investigated with fMRI. The most investigated is the situation-focused strategy (REAPPsit), which involves reinterpreting the meaning of the emotional actions or events presented, in order to reduce the emotional response (e.g., seeing a diseased person and thinking the person will get better). The other type is the self-focused reappraisal strategy (REAPPself), which is also known as distancing (e.g., Ochsner et al., 2004), i.e., adopting the role of a detached third-person observer during the presentation of the emotional stimuli (e.g., thinking that the presented stimuli are randomly seen in a newspaper). Ochsner et al. (2004) compared the neural response of both reappraisal strategies, linking regions of the medial prefrontal cortex (mPFC) to REAPPself and more lateral prefrontal cortex regions (lPFC) to REAPPsit (Ochsner et al., 2004). Furthermore, it has been discussed that the ability to assume a subjective distance to emotional cues implies a change in the perceived self-relevance of emotion-inducing objects, which is associated with a right-lateralized, mPFC activity (Kalisch et al., 2005; Ochsner et al., 2005; Ochsner and Gross, 2008; Leiberg et al., 2012). On the other hand, the act of reinterpret negative events might require a more left-lateralized, lPFC involvement because of a highly verbal, externally focused control processing during REAPPsit (see also Ochsner et al., 2012).

In order to examine whether specific regions in the PFC are crucial for effective reappraisal of negative stimuli, brain lesion studies might be an excellent extension to fMRI data, as they do not only reflect which areas are associated with a given ability, but also show which regions are critical for function integrity (Rorden and Karnath, 2004). Furthermore, the need for this type of studies on ER has been highlighted in the past (firstly addressed by Beer and Lombardo, 2007). However, to date there have been only two studies investigating reappraisal performance after acquired brain damage. One recent case-study showed that emotional reappraisal is impaired after a left PFC stroke lesion (Salas et al., 2013). Another recently published study of the same author investigated behavioral data of reappraisal difficulty and productivity in brain injured patients. Results showed that cognitive skills, such as inhibition and verbal fluency might be strongly associated with the generation of reappraisals. Still, the reappraisal ability was not addressed (Salas et al., 2014). Therefore, the goal of the current study was to explore the reappraisal ability in brain-injured patients, and to infer which area of the PFC impairs this ability the most. For this purpose, considering that the effects of a brain lesion might impair the manipulation of thoughts (as held by Salas et al., 2014), we chose to hold the reappraisal strategy constant and instruct the REAPPself strategy. First, this strategy has been shown to be more effective than REAPPsit at overall reduction of affective responding (Shiota and Levenson, 2012). Second, REAPPself might be less difficult than REAPPsit for the brain-damaged patients, as REAPPsit might require more cognitive abilities for the spontaneous generation of alternative reinterpretations and involves more contextual encoding of stimuli than REAPPself (Ochsner et al., 2004, 2012; Ochsner and Gross, 2008). Furthermore, REAPPself might be a relevant strategy for brain-damaged patients, given the widely recognized importance of self-distancing for adaptive coping with autobiographical negative events (Ayduk and Kross, 2008, 2010).

To explore which lesion location is most likely to impair REAPPself, we first ran an exploratory voxel-based lesion-symptom mapping analysis (VLSM; for further explanation of this method see Bates et al., 2003; Rorden et al., 2007) in 20 patients with single brain lesions. We hypothesized that SFG regions, particularly regions near the mPFC, would be indispensable for REAPPself ability (Ochsner et al., 2004), as this region has anatomically been defined as a mediator between the lateral PFC and amygdala regions (Johnstone et al., 2007; Ray and Zald, 2012). Second, we predicted that subjects with a lesion in the areas highlighted by the VLSM analysis, defined as region-of-interest (ROI), would have deficits on REAPPself, whereas patients with a lesion sparing these ROI would show a better REAPPself ability. Third, we ran a regional voxel-based-morphometry (VBM; Ashburner and Friston, 2000) analysis with structural data of healthy participants expecting to find significant associations between ROI gray matter intensities and REAPPself function. All participants completed cognitive and affective screening tasks in order to characterize potential deficits of the patients. Here, we also aimed to explore which cognitive functions are more impaired in the group with a lesion in the ROI compared to the other groups.

## Methods and materials

### Participants

A total of 27 patients with lesions in different parts of the cortex, but predominantly affecting the frontal lobes, and 23 healthy volunteers were assessed. The patients were recruited either from the department of Neuro-Oncology of the University Hospital of Heidelberg, Germany, or from self-help groups in the community. The inclusion criterion was the presence of a lesion involving well-defined parts of the cortex with definable and segmentable margins in the T_2_ weighted FLAIR (fluid-attenuated inversion recovery) sequence, assessed by an experienced radiologist. For all analyses, a potential edema zone was, if present, taken into account. Patients with multifocal brain lesions, previous history of head trauma or neurological disorders independent of brain injury, clinically detectable aphasia symptoms as well as presence of serious functional impairments (i.e., patients with a Karnofsky index below 80%; Clark and Fallowfield, 1986) were excluded. Healthy control participants (15 women; *M* age = 39.65 ± 11.23) had no history of neurological or psychiatric disorders. No control participant was taking psychoactive drugs. They were recruited through advertisements posted in newspapers, and were matched as closely as possible to the patients for sex and age.

Nineteen patients with an acquired brain tumor and one patient with a cyst met the inclusion criterion. The diagnosis of patients with brain tumors has been histopathologically confirmed either by operation (*n* = 17) or by biopsy (*n* = 2) in agreement with the WHO staging system (Kleihues and Sobin, 2000). Seven patients had to be excluded, due to non-corrected vision problems (*n* = 1), multifocal lesions (*n* = 5) and severe microangiopathy (*n* = 1). The remaining 20 patients took part in the analysis (11 women, *M* age = 45 ± 10.04 years; *M* lesion volume = 35.27 ± 32.19 cm^3^). Of 19 brain tumor patients, 10 had single low-grade tumors (WHO°II) and 9 had single high-grade tumors (WHO°III-IV). Patients with brain tumors received radio- and/or chemotherapy. Brain tumor patients were tested at least 1 year after biopsy or maximum safe resection (*M* biopsy/resection = 3.95 ± 4.20 years, range = 1–16 years), and the whole group was tested at least 2 years since lesion onset (*M* onset = 5.00 ± 3.96 years, range = 2–15 years). Sixty-five percent of the 19 patients with brain tumors had no evidence for tumor recurrence and 35% had tumor recurrence in the same lesion site. Taking perifocal edemas into account, no lesion was bigger than 110 cm^3^ (see Table 1 for more detailed information about etiology, lesion laterality and tumor location).

**Table 1:** **Brain-damaged patient sample**.

**Nr.**	**Gender (m/f)**	**Age**	**Education level (1–8)**	**Etiology**	**Years post onset**	**Lesion side**	**Lesion location**	**Severity**	**Lesion volume (cm^3^)**
1	f	59	4	Resected Oligodendroglioma	10	Right	Frontobasal	WHO°III	1.19
2	f	42	4	Resected Meningioma	15	Left	Frontotemporal	WHO°II	3.74
3	f	65	8	Resected Glioblastoma	1	Right	Frontoparietal	WHO°IV	33.02
4	f	30	8	Resected Astrozytoma	2	Left	Parietal	WHO°II	43.92
5	f	38	8	Resected Oligodendroglioma	5	Left	Frontal	WHO°III	76.82
6	m	46	8	Resected Oligoastrozytoma	7	Right	Frontotemporo-parietal	WHO°III	70.02
7	m	46	8	Resected Oligoastrozytoma	6	Left	Frontal	WHO°III	39.98
8	f	40	3	Astrozytoma	2	Right	Frontal	WHO°II	5.67
9	m	55	8	Resected Oligodendroglioma	3	Bilateral	Frontal	WHO°III	90.09
10	f	52	6	Resected Glioblastoma	2	Right	Frontal	WHO°IV	46.53
11	f	61	2	Resected Glioblastoma	2	Left	Frontal	WHO°IV	14.56
12	m	32	2	Astrozytoma	2	Left	Frontal	WHO°II	9.58
13	m	32	8	Resected Astrozytoma	3	Left	Frontoparietal	WHO°II	11.61
14	f	45	8	Resected Oligodendroglioma	2	Right	Frontal	WHO°III	49.32
15	f	51	7	Resected Astrozytoma	4	Right	Frontal	WHO°II	15.74
16	m	40	2	Astrozytoma	4	Right	Insular	WHO°II	4.44
17	m	43	2	Astrozytoma	2	Right	Frontal	WHO°II	20.47
18	m	36	2	Resected Astrozytoma	12	Left	Temporal	WHO°II	55.20
19	m	49	2	Resected Astrozytoma	11	Bilateral	Frontotemporo-parietal	WHO°III	110.45
20	f	37	8	Cyst involving cortex	4	Right	Frontal	–	3.08

*f, female; m, male*.

All participants were informed about the risks of the study and signed a written informed consent prior to participation. This research was conducted with the approval of the ethical board of the University of Heidelberg according to the declaration of Helsinki. All participants were paid after finished assessment.

### Neuropsychological and affective assessment

All participants completed a brief screening of cognitive abilities, which included measures of cognitive flexibility (Trail Making Task A,B; Tombaugh, 2004); fluid intelligence using the *Culture-Fair-Intelligence-Test* (CFT-20; Cattell, 1960; Weiss, 1998); memory performance and processing speed (measured by the cognitive screening of the German Aphasia-Check-List ACL; Kalbe et al., 2002, 2005) and behavioral inhibition (Go/Nogo task from the German Attention Test Battery TAP; Zimmermann and Fimm, 2002). The Beck Depression Inventory (BDI-II; Kuhner et al., 2007) was included for the assessment of depressive symptoms (for more information see the Supplementary Material).

### Stimulus material

A set of 20 negative and 20 neutral pictures was selected from the *International Affective Picture System* (IAPS; Lang et al., 2005). The selected negative pictures mainly contained unpleasant images of injured or mutilated persons, violent situations and diseases (*M* arousal = 6.91 ± 0.30; *M* valence = 2.01 ± 0.53), while the neutral pictures showed mainly ordinary home objects (*M* arousal = 2.69 ± 0.52; *M* valence = 4.98 ± 0.23). Neutral and negative pictures differed significantly in arousal [*t*_(26)_ = −22.96; *p* < 0.001] and valence ratings [*t*_(30.58)_ = 31.59; *p* < 0.001)].

### REAPPself task

**Figure 1:**
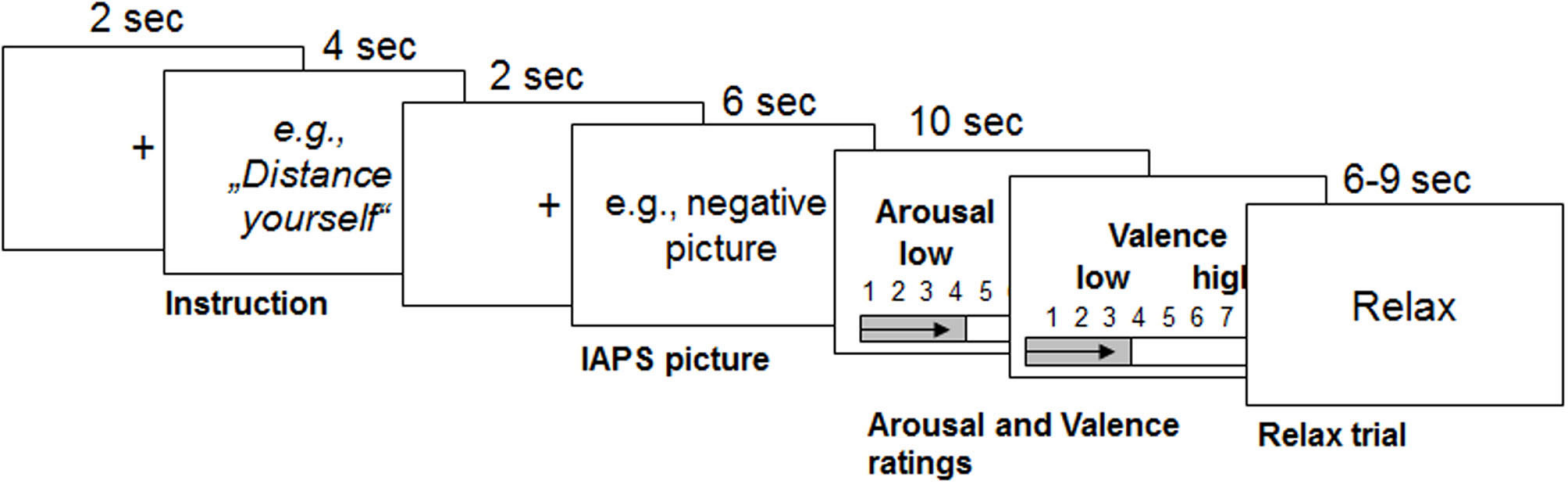
**Graphical description of the reappraisal task used with the durations of each trial (modified from Ochsner et al., 2004)**. Emotions were triggered by IAPS pictures after the instruction was presented for 4 s. From this point on, participants had to either only look at the picture or down-regulate the triggered emotions for 6 s. Then they had to rate their emotional arousal and valence for 5 s each. Afterwards, participants relaxed for 6–9 s.

The task consisted of three conditions: the neutral condition, in which participants had to watch neutral pictures (LNeu); the negative condition, in which participants had to watch negative affective pictures (LNeg); and the REAPPself condition, in which participants had to decrease the triggered negative emotions by means of REAPPself during negative picture presentation (Dec). Sixty pseudo-randomized trials were presented using the *Presentation experiment driver* (www.neurobs.com). A typical trial started with a white fixation cross on a black background, which was presented for 2 s. Afterwards, the instruction was presented for 4 s: either “LOOK” (solely look at the picture without trying to manipulate the induced emotion) or “DISTANCING” (e. g. “It is a newspaper picture, and I am not involved”). Then, a brief fixation cross was presented again, followed by the negative emotional picture, presented for 6 s. Participants had to process the pictures according to the instructions. Self-assessment manikin (SAM Ratings; Bradley and Lang, 1994) were presented directly afterwards. Here, participants had to spontaneously rate the amount of emotional arousal as well as how displeasing the emotion induced by the previously presented picture was (valence); patients rated on a 1–9 scale. As the task was originally design for fMRI, a random jitter (6–9 s) relax trial appeared on the screen afterwards (Amaro and Barker, 2006). For a graphical description of the experimental order see Figure 1.

### Procedure

The assessment was separated into two sessions, with less than a week between assessments. The first session comprised cognitive and affective assessment, whereas the second session involved the magnetic resonance imaging (MRI) scan and the REAPPself fMRI-task. In the current article, only behavioral results are reported.

First, the examiner provided the reappraisal task instructions in a written form. Then, participants were confronted with negative pictures of the IAPS while becoming clear instructions of REAPPself: to decrease the induced negative emotion by perceiving the content of the stimuli in a detached, third-person perspective (as viewing the picture in a newspaper; e.g., Ochsner et al., 2004; Lang et al., 2012). Thus, participants had to think, for example, that the event showed in the picture occurs in a place far away or is randomly seen in the newspaper. Further, they informed the examiner by saying out loud how they detached. Subsequently, participants were trained in REAPPself by performing three practice trials. The session ended after ensuring that the participant understood the task procedure and applied REAPPself properly. In the second session, four more practice trials were conducted in the MRI scanner, in order to ensure that the participants felt comfortable. After the investigator was sure that the task was understood, participants began with the task assessment, which was separated into two runs of 30 trials each.

### Structural imaging recordings and lesion analysis

Structural images were obtained on a 3 Tesla MRI scanner system (MAGNETOM Trio, Siemens Medical Systems, Erlangen, Germany) equipped with a 32-channel head coil. All brains were visualized with high resolution scans, which were acquired using a T1-weighted flash 3D sequence (*TR* = 1680 ms; *TE* = 2.6 ms; voxel size = 1.1 × 1.1 × 1.1 mm). Individual edema and tumor tissue were traced from T2-weighted FLAIR anatomical scans (*TR* = 9000 ms; *TE* = 95.0 ms; voxel size = 0.9 × 0.9 × 4.0 mm) by a radiologist blind to task performances using MRIcron software (Rorden et al., 2007). Using a procedure endorsed by Crinion et al. (2007), the T2 scans with the identified lesions were co-registered to the T1-weighted scans. Finally, the T1 scans were normalized to standardized MNI-space via *Unified segmentation and normalization* of the MATLAB toolbox *Statistical Parametrical Mapping* (SPM8; Crinion et al., 2007; Seghier et al., 2008). The Montreal Neurological Institute (MNI) brain standardized lesions were used to estimate lesion sites on aal templates of the MRIcron software (www.mricro.com/mricron) and to create lesion overlap images.

### VLSM analysis

**Figure 2:**
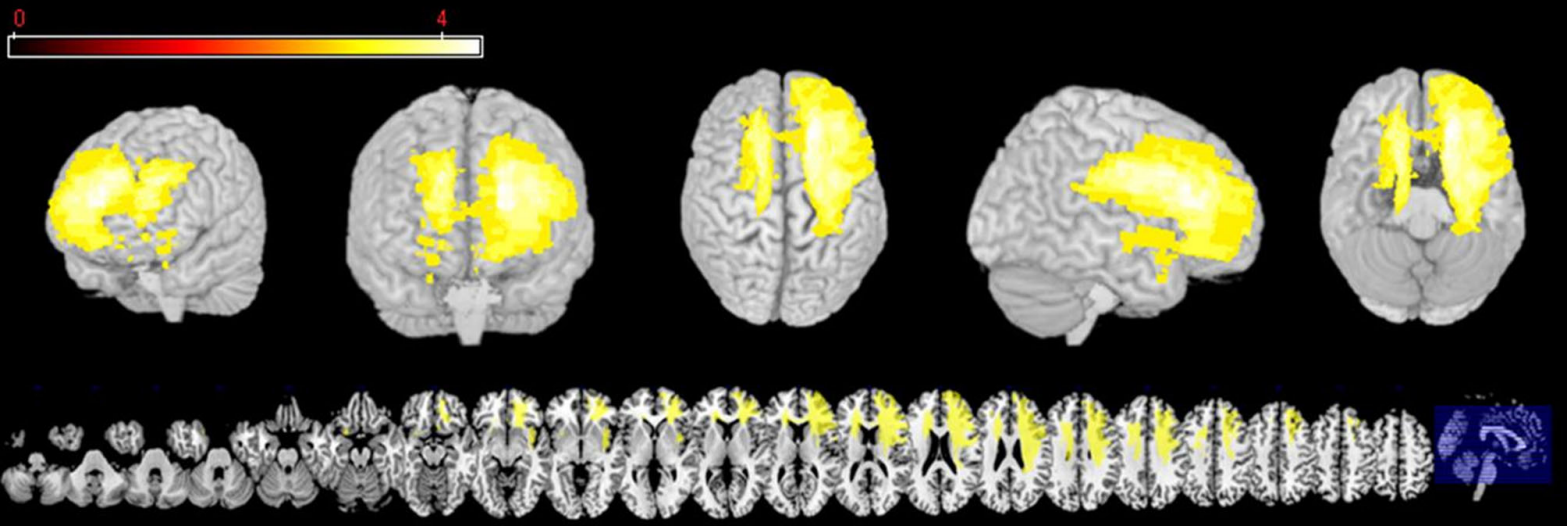
**Three-dimensional and multislice views of voxels (yellow), where there is sufficient statistical power to detect an effect of lesion on behavior**.

The first analysis was run in VLSM (Bates et al., 2003) in order to explore which regions are associated with impairments of REAPPself ability. The input variables were the operationalization of the REAPPself ability (i.e., the amount of down-regulation), which comprised the subtractions in arousal and valence ratings from “LOOK” and “DISTANCING” conditions (LNeg-Dec). Then, a series of Brunner-Munzel (BM) *t*-tests at every voxel were run in order to compare the input variables in patients with and without a lesion in the voxel. Based on the results (*p* < 0.05), a colorized map was generated, showing which lesioned region/s is/are associated with poorer performance. For instance, if patients with a lesion in specific voxels show significantly poorer REAPPself ability, then the region in which these specific voxels are located would be visualized in the statistical map. We also generated a map to determine the distribution of statistical power for our sample, based on an effect size of 0.8 (Kimberg et al., 2007) and an alpha level of 0.05, which shows voxels with enough power to detect significant differences. As shown in Figure 2, mostly right PFC and left superior PFC areas had adequate power. For instance, predictions for the VLSM analysis were restricted to these regions. In order to prevent spurious results, solely voxels in which a minimum of three patients was affected were included (analogously to Tsuchida et al., 2010; Tsuchida and Fellows, 2012). Significant results were overlaid to an MRIcron template (http://www.mccauslandcenter.sc.edu/mricro/mricron/) in order to identify involved Brodmann areas (BA).

### VLSM region-of-interest (ROI) groupwise comparison

The second analysis depicts group comparisons between patients presenting a lesion in the overlap area calculated by VLSM (ROI), patients with a lesion sparing the ROI (IntactROI), and the healthy control (HC) group. Mean arousal and valence ratings were included as dependent variables in 3 *groups* (ROI, IntactROI, HC) × 2 *tasks* (LNeg, Dec) repeated measures ANOVA, followed by *post-hoc* Tukey HSD pair-wise comparisons. For relevant control variables, we used the Kruskal-Wallis-Test. Variables differing significantly between groups were included as covariate in the ANOVA.

### VBM analysis

Images for control subjects were preprocessed for VBM analysis using DARTEL Toolbox and followed procedures previously described (Ashburner and Friston, 2000). Following, the modulated images were smoothed with a Gaussian 12 mm full-width half-maximum kernel as suggested in other reports (Good et al., 2001) and normalized to the MNI standard space. Finally, these images were analyzed within different general linear models in SPM-8 2nd level analyses (http://www.fil.ion.ucl.ac.uk/spm/software/spm8). These consisted of multiple linear regressions accounting for total intracranial volume, age and gender as non-interest or nuisance covariates. First, two whole brain analyses were performed: (1) for arousal differences (LNeg-Dec) and (2) for valence difference scores (LNeg-Dec) of REAPPself ability. The analyses were performed and examined at *p* < 0.001, two-tailed uncorrected threshold. Second, in order to assess the specific regional pattern of gray matter involved in each domain, two linear regressions were performed within the VLSM depicted ROI of the patients group as a small volume correction.

## Results

### Sociodemographic, neuropsychological, and affective assessment

The independent *t*-test revealed no significant differences between healthy controls (*n* = 23) and patients (*n* = 20) in age [*t*_(41)_ = −1.64, *p* = 0.11], but significant differences in educational level [*t*_(26.81)_ = 2.37, *p* = 0.03], depression [*t*_(20.13)_ = −5.05, *p* < 0.001] and fluid intelligence scores [*t*_(37)_ = 2.77, *p* = 0.01] (see Table 2). Regarding the neuropsychological screening, patients differed significantly from the healthy control performance in processing speed assessed by phonemic verbal fluency [*t*_(41)_ = 3.49, *p* = 0.001], short-term memory [*t*_(29.97)_ = 2.92, *p* = 0.007], behavioral inhibition assessed by the number of Go/NoGo errors [*t*_(19.87)_ = −2.70, *p* = 0.01] and cognitive flexibility [*t*_(31.51)_ = −2.49, *p* = 0.02]. The cognitive profile of the patient group is consistent with several descriptions of performance on individuals with frontal lobe lesions (see Table 3; Dimitrov et al., 2003; Niki et al., 2009; Rodriguez-Bailon et al., 2012).

**Table 2:** **Demographic variables of brain-damaged patients and healthy controls**.

**Variables**	**Patients (*n* = 20, 11 f)**	**Controls (*n* = 23, 15 f)**	***p***	**Effect size (*d*)**
Age	45 (10.04)	39.65 (11.23)	0.11	0.50
Educational level (1–8)	5.4 (2.74)	7 (1.35)	0.03	0.74
Depressive symptoms (BDI)	12.67 (8.44)	2.17 (2.88)	<0.001	1.67
Fluid IQ (CFT-20)	109 (12)	118.47 (9.12)	0.01	0.89

*BDI, Becks Depression Inventory; CFT, Culture Fair Intelligence Test; f, female*.

**Table 3:** **Summarized performance results on selected neuropsychological screening tests for brain-damaged patients and healthy controls**.

**Mean (*SD*)**				
**Variables**	**Patients (*n* = 20)**	**Controls (*n* = 23)**	***p*^a^**	**Effect size (*d*)**
**PROCESSING SPEED (ACL)^b^**				
Phonemic verbal fluency	12.05 (3.07)	15.74 (3.76)	0.001	1.13
Semantic verbal fluency	17.95 (5.21)	21 (4.99)	0.06	0.60
*Cognitive Flexibility* (TMT B-A)	35.39 (16.76)	24.48 (10.74)	0.02	0.78
**MEMORY RECALL (ACL)^b^**				
Inmediate recall	4.7 (1.22)	5.61 (0.72)	0.01	0.91
Delayed recall	4.9 (1.33)	5.52 (0.85)	0.08	0.56
**BEHAVIORAL INHIBITION (GoNoGo)**				
Median reaction time	401.06 (75.86)	420.09 (53.12)	0.36	0.29
Comission errors	1.72 (2.05)	0.36 (0.66)	0.01	0.89
Omissions	0.67 (1.85)	0.46 (0.21)	0.17	0.16
Outliers	0.28 (0.46)	0.27 (0.55)	0.98	0.02

a *Independent t-test results*,

b *Cognitive assessment section of the Aphasia Check List test battery; SD, standard deviation; ACL, Aphasia Check-List; TMT, Trail Making Task*.

### Group comparisons in emotional reactivity and regulation

In order to examine the differences on emotional reactivity, we subtracted the arousal and valence ratings of LNeu conditions from LNeg. The independent *t*-test with both groups showed that on average, patient and control groups did not significantly differ in emotional reactivity, neither for arousal [*t*_(29.98)_ = 0.72, *p* = 0.48], nor for valence [*t*_(41)_ = −0.52, *p* = 0.61].

**Figure 3:**
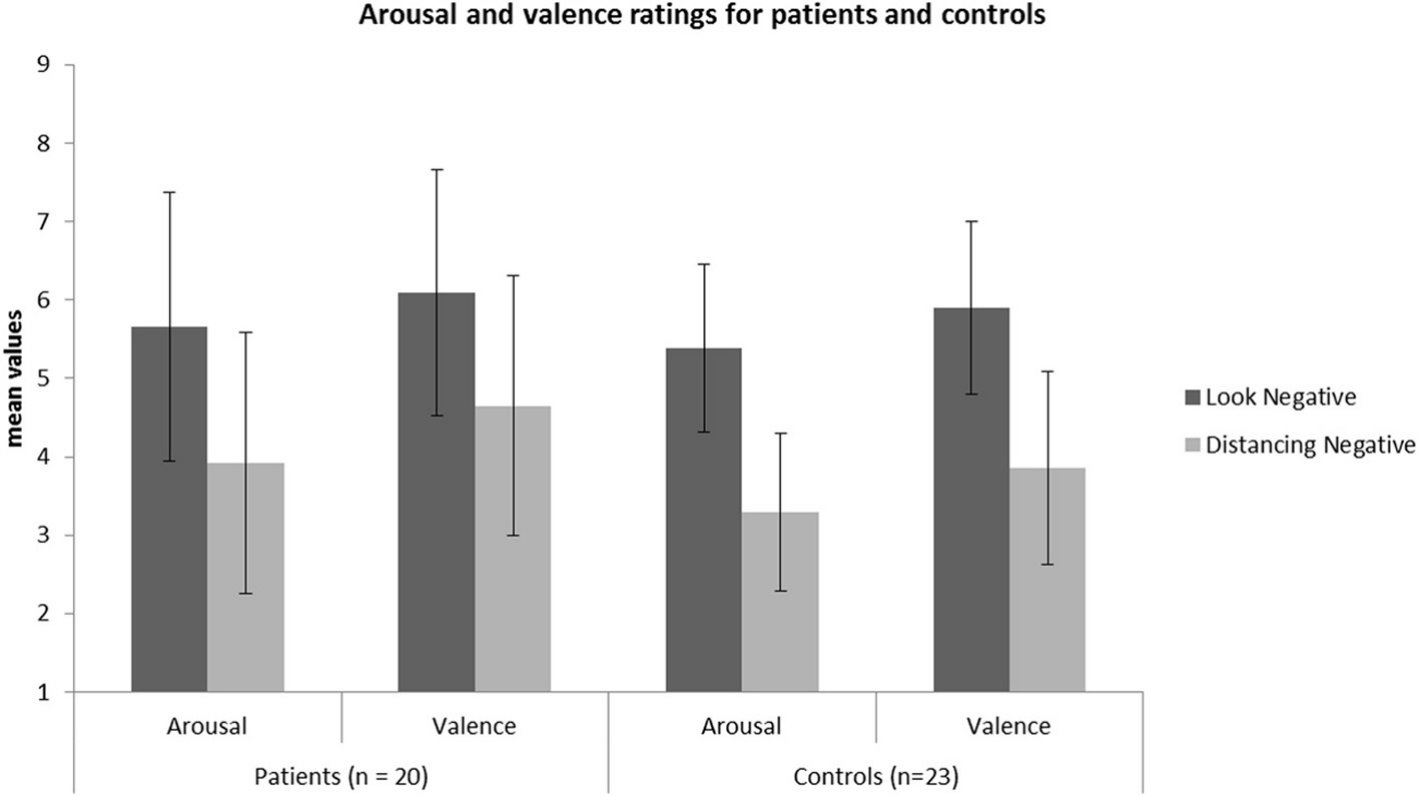
**Mean values of the arousal and valence ratings for the patient (*N* = 20) and HC (*N* = 23) sample in each condition**.

Following, to investigate whether patients and HC differed significantly in REAPPself ability, we ran two repeated-measures ANOVAs with arousal and valence ratings of LNeg and Dec *tasks* as within-subject factors, and *group* (patients vs. HC) as between factors. Given that depressive symptoms (arousal: *r* = 0.43; *p* = 0.006, valence: *r* = 0.47; *p* = 0.002), fluid intelligence (arousal: *r* = −0.34; *p* = 0.04), short-term/immediate memory (arousal: *r* = −0.35; *p* = 0.02, valence: *r* = −0.36; *p* = 0.02) and inhibition deficits (arousal: *r* = 0.31; *p* = 0.05) significantly correlated with arousal and valence ratings in the Dec condition, we included each of these variables as covariates in an ANOVA design of arousal and valence ratings, and tested their significance regarding interaction effects with the dependent variables. It is important to mention that the variables did not correlate with the ratings in the LNeg condition. No significant interaction effects with the dependent variables were found. Only the depression (BDI) scores reached significant main effects as a covariate for the arousal [*F*_(1, 38)_ = 5.57; *p* = 0.02] and valence [*F*_(1, 38)_ = 7.27; *p* = 0.01] ratings. So, controlling for depression, there was no main effect for group [arousal: *F*_(1, 38)_ = 0.54, *p* = 0.47; valence: *F*_(1, 38)_ = 0.70, *p* = 0.41] or interaction effects *group* x *task* [arousal: *F*_(1, 38)_ = 0.23, *p* = 0.63; valence: *F*_(1, 38)_ = 1.41, *p* = 0.24], whereas effects of task were highly significant [arousal: *F*_(1, 38)_ = 63.18, *p* < 0.001; valence: *F*_(1, 38)_ = 58.23, *p* < 0.001]. Thus, all participants were able to down-regulate the emotional valence of the pictures (see Figure 3). Interestingly, by excluding the depression covariate from the analysis, the valence ratings showed significant interaction effects *group* x *task* [*F*_(1, 41)_ = 4.38, *p* = 0.04], reflecting that the valence-related down-regulation was influenced by depressive symptoms.

### Brain-damaged patients' gray matter results

**Figure 4:**
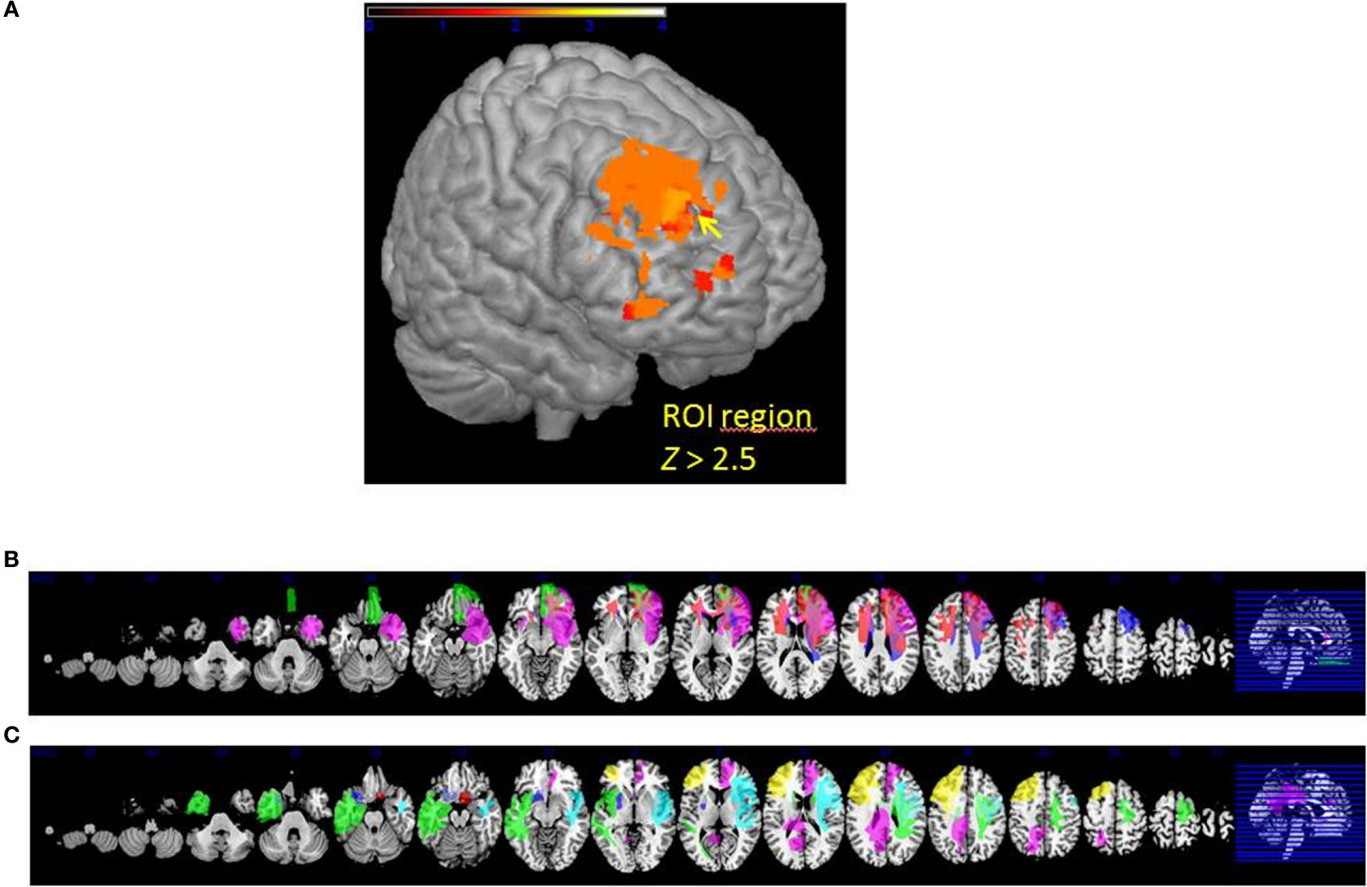
**(A)** VLSM results: brighter regions showing more significant effects in dorsal regions of the PFC (cutoff *Z* > 2.5) **(B)** multislice view of lesion overlays of ROI group including edema zone (*N* = 5) **(C)** multislice view of the IntactROI group (*N* = 15).

The VLSM analysis of the arousal-related REAPPself ability scores showed the involvement of a region in SFG overlapping the right dlPFC (BA9) and the right dACC (BA32). The valence-related REAPPself ability did not show any cortex involvement. The statistical map generated by the BM-test on each voxel is shown in Figure 4A. The color scale indicates BM-test *Z*-scores. It is important to mention that no voxel survived correction for multiple comparisons using conventional false discovery rate (FDR) thresholds (e.g., Tsuchida et al., 2010). However, the power map in Figure 2 shows that this region in the right SFG had adequate power to detect effects at the uncorrected threshold depicted in Figure 4A. Nevertheless, the statistical map of the BM-test should be interpreted with caution, because of the risk of false-positive findings. We conducted a ROI-based analysis to further analyze the functional effects of lesions in the depicted voxels (Kimberg et al., 2007). For this purpose, we focused on the brighter regions showing results thresholded with a cut-off value of *Z* > 2.5.

### ROI groupwise comparison

The patient sample was subsequently divided into two groups. The ROI group consisted of five patients presenting a lesion overlapping the region of the computed VLSM analysis (*N* = 5; see Figure 4B). The IntactROI group consisted of patients presenting a lesion sparing this region (*N* = 15; see Figure 4C). Compared with the HC in a Kruksall-Wallis-test, groups showed no significant differences in age (*p* = 0.22), education level (*p* = 0.21), or lesion volume (*p* = 0.13). However, groups differed significantly in depressive symptoms (*p* < 0.001; ROI>IntactROI>HC), fluid intelligence (*p* = 0.01; ROI<IntactROI<HC), phonemic verbal fluency (*p* = 0.01; ROI<IntactROI<HC), short-term memory (*p* = 0.02; ROI<IntactROI<HC), and inhibition deficits, as assessed by the number of Go/Nogo errors (*p* = 0.002; ROI>IntactROI>HC). Interestingly, the covariance main effect of depressive symptoms was significant for valence rating scores [*F*_(1, 37)_ = 4.27; *p* = 0.05], but not for arousal scores [*F*_(1, 37)_ = 2.71; *p* = 0.11]. However, no significant interaction effects BDI x *task* [arousal: *F*_(1, 38)_ = 0.001, *p* = 0.98; valence: *F*_(1, 37)_ = 0.15, *p* = 0.71] and no significant correlations with REAPPself ability scores were found (arousal: *r* = −0.20, *p* = 0.21; valence: *r* = −0.30, *p* = 0.06), so that they were not included in the analysis. In addition, the Kruksal-Wallis test showed that the groups did not significantly differ in emotional reactivity scores (LNeg-LNeu; arousal: *p* = 0.54; valence: *p* = 0.87).

**Figure 5:**
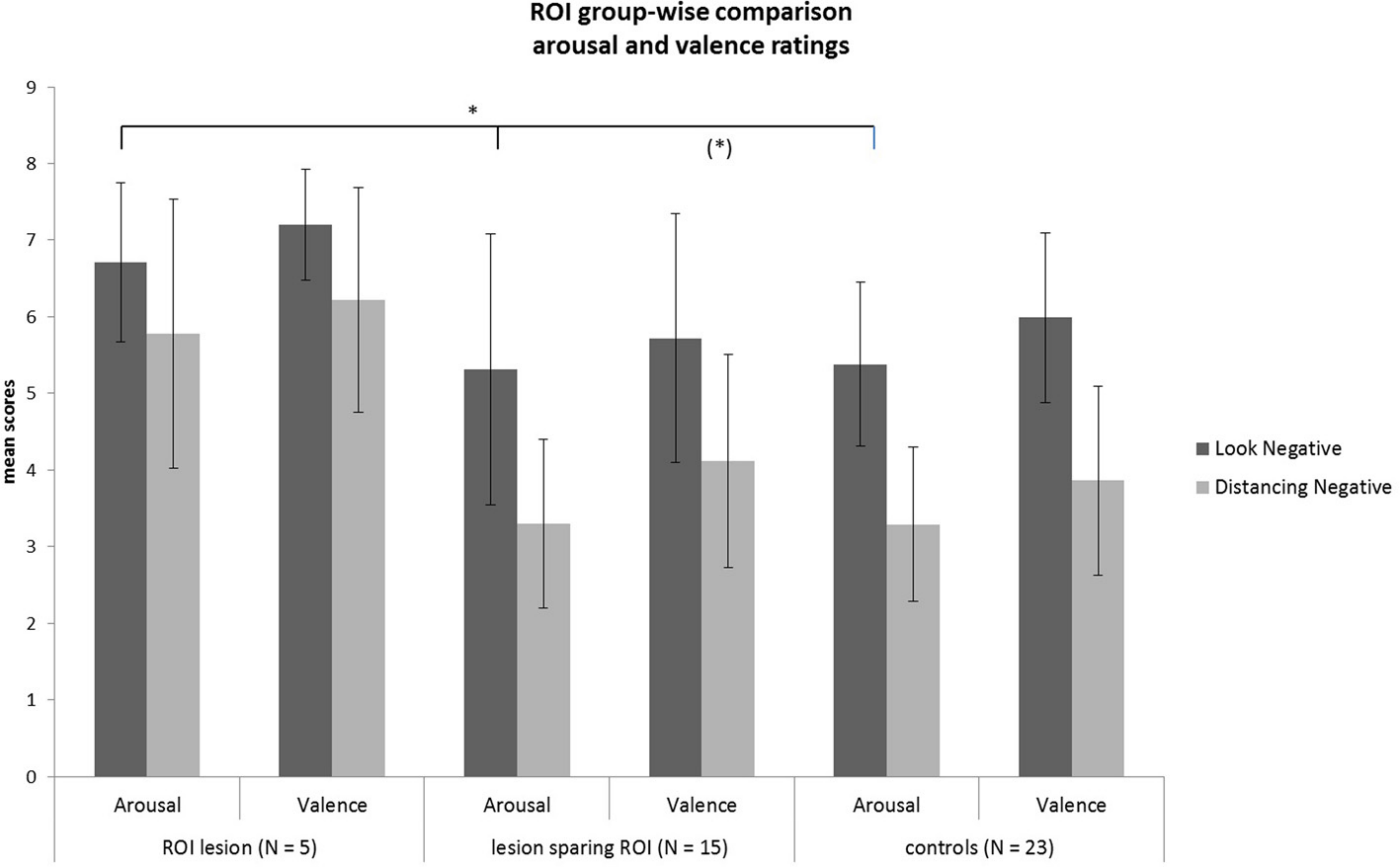
**Subjective ratings of arousal and valence after look and decrease conditions for each group**. There was a significant group difference of decrease scores showing that the ROI-group was the less successful in down-regulating negative emotions. ^*^p < 0.05.

Rating values demonstrated significant overall main effects of *group* [arousal: *F*_(2,40)_ = 6.07, *p* = 0.005; valence: *F*_(2,40)_ = 4.93, *p* = 0.01] and *task* [arousal: *F*_(1, 40)_ = 94.35, *p* < 0.001; valence: *F*_(1, 40)_ = 80.65, *p* < 0.001]. A significant interaction effect *group* × *task* of arousal ratings [*F*_(2, 40)_ = 3.28, *p* = 0.05] demonstrated that patients with a lesion in the ROI could not down-regulate arousal induced by negative emotions in the same manner as the two other control groups, as shown in Figure 5 (see also Tukey comparisons in Table 4). In other words, negative arousal in the Dec condition was significantly higher for the ROI group compared to the other control groups. In addition, valence *group* × *task* interactions were marginally significant [*F*_(2, 40)_ = 3.07, *p* = 0.06]. Results of Tukey's test for multiple comparisons revealed that arousal and valence rating scores were significantly higher for the ROI group than the two other groups, which did not differ significantly in arousal and valence scores (*p* = 0.99; see Table 5). These results indicate a more successful down-regulation of negative emotions for the two control groups (REAPPself ability), but less successful for the ROI group. Regarding the valence ratings, this difference might be significantly influenced by the amount of depressive symptoms of the ROI group.

**Table 4:** **Multiple comparisons (Tukey HD *post-hoc* tests) of arousal and valence ratings of ROI injured, ROI intact patients, and healthy controls**.

		**Arousal ratings**			**Valence ratings**		
**I Group**	**II Group**	**Mean difference I-II**	**St. error**	***p***	**Mean difference I-II**	**St. error**	***p***
ROI group	Intact ROI	1.94	0.59	0.007	1.79	0.63	0.02
	HC	1.91	0.58	0.005	1.83	0.59	0.01
Intact ROI	ROI group	−1.94	0.59	0.007	−1.79	0.63	0.02
	HC	−0.032	0.38	0.99	0.04	0.40	0.99
HC	ROI group	−1.91	0.57	0.005	−1.83	0.59	0.001
	Intact ROI	0.032	0.38	0.99	−0.04	0.40	0.99

**Table 5:** **Whole brain Patterns of GM intensity correlated with task performance in controls**.

		**Coordinates**				
**Region**	**BA**	***x***	***y***	***z***	***F* peak**	***p* (unc)**
**A. AROUSAL**						
Superior frontal gyrus R	9	18	34.5	40.5	20.97	<0.001
Cerebellum Crus 2 R		7.5	−90	−36	20.39	<0.001
Pallidum L		−13.5	6	0	16.79	<0.001
Mid temporal gyrus L	22	−52.5	−49.5	0	12.54	<0.001
Posterior Insula L	13	−34.5	−28.5	16.5	12.19	<0.001
Superior frontal gyrus L	9	−16.5	43.5	24	12.19	<0.001
Rolandic Operculum R	13	43.5	−9	22.5	11.84	<0.001
Insula L	13	−36	−9	19.5	11.83	<0.001
Cerebellum Crus 1 L		−36	−58.5	−37.5	11.80	<0.001
**B. VALENCE**						
Cerebellum 8 L		−27	−43.5	−54	15.82	<0.001
Mid frontal gyrus L	10	−34.5	49.5	16.5	15.80	<0.001
Cerebellum Crus 2 R		7.5	−88.5	−34.5	15.43	<0.001
Mid temporal gyrus R	21	42	−64.5	6	14.74	<0.001
Cerebellum Crus 10 R		22.5	−40.5	−46.5	14.04	<0.001
Putamen L		−16.5	3	−7.5	14	<0.001
Inferior parietal lobule R	7	30	−54	39	12.38	<0.001
Inferior frontal gyrus *Pars Triangularis* L	46	−48	19.5	9	12.08	<0.001
Superior temporal gyrus R	22	60	−31.5	10.5	11.97	<0.001
Superior frontal gyrus R	9	22.5	−22.5	58.5	11.73	<0.001

### Healthy controls' gray matter results

#### Whole brain analyses

**Figure 6:**
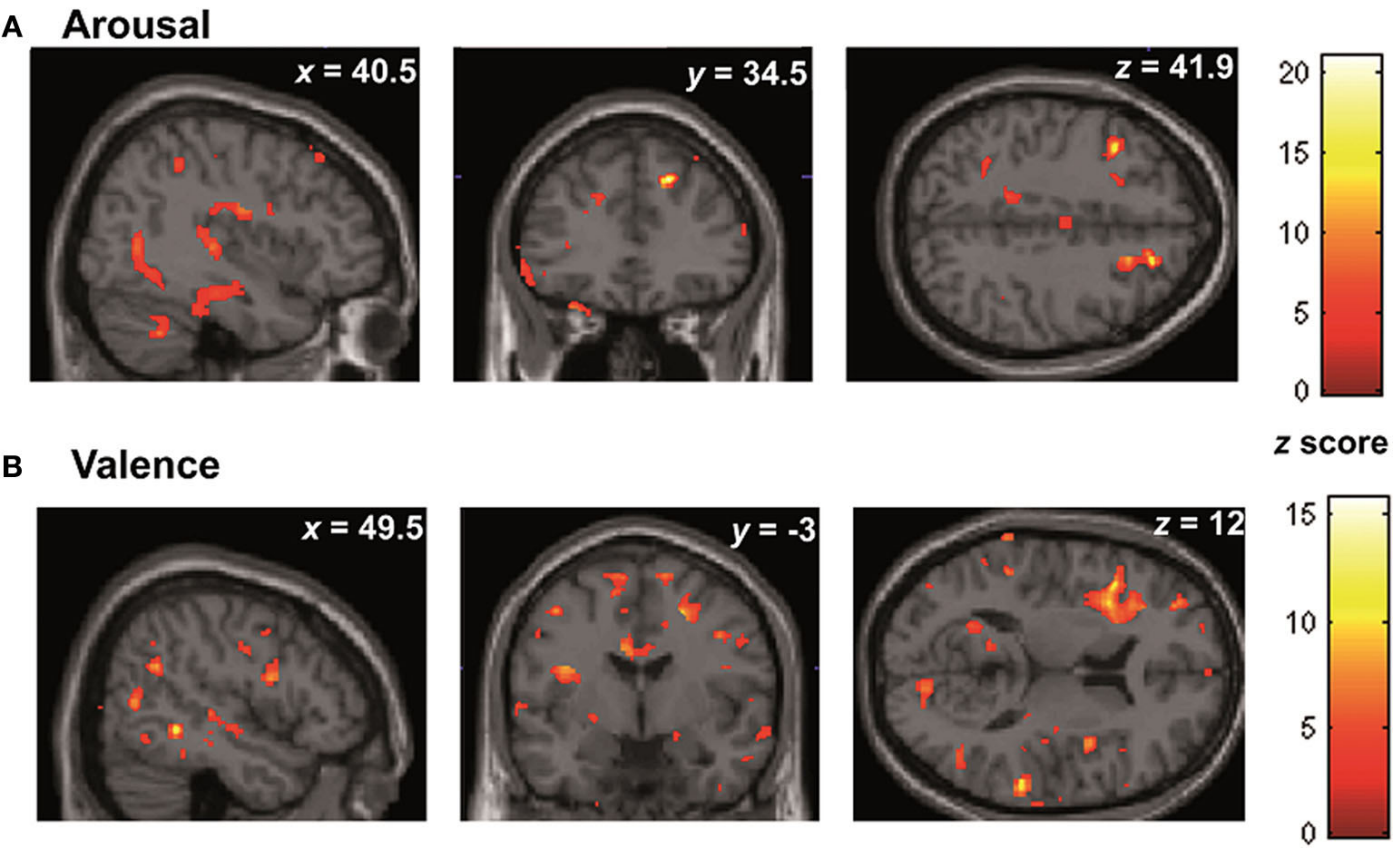
**Whole brain patterns of gray matter intensities correlated with task performance in controls. (A)** Arousal rating differences **(B)** Valence rating differences.

In the whole brain analyses, it was found that REAPPself ability scores (LNeg-Dec) had a positive correlation with gray matter of a series of cortical and subcortical structures in the right and left hemispheres: at the frontal lobe, more specifically the right SFG (BA 9-32; see Figure 6A), left insula, basal ganglia and mid temporal gyrus, and bilateral cerebellum (See Table 5A for MNI coordinates). For the valence (LNeg-Dec) domain, this relation appeared at the left SFG (BA 9-32; see Figure 6B), right SFG, left mid and inferior frontal gyri, temporal cortex, parietal cortex, basal ganglia and cerebellum (See Table 6B for MNI coordinates). No significant negative correlations were found.

**Table 6:** **Patterns of regional GM intensity correlated with task performance in controls**.

		**Coordinates**				
**Region**	**BA**	***x***	***y***	***z***	***F* peak**	***P* (FDR-cor)**
**A. AROUSAL**						
Superior frontal gyrus R	9	21	36	39	2148	<0.001
		15	28.5	37.5	1135	<0.001
Anterior cingulate R	32	13.5	28.5	27	1063	<0.001
**B. VALENCE**						
Superior frontal gyrus R	9	21	39	37.5	1617	<0.001
		15	28.5	37.5	856	<0.001
Anterior cingulate R	32	13.5	28.5	27	996	<0.001

*VBM, voxel-based morphometry; BA, Brodmann area; R, right; L, left; GM, gray matter; FDR, false-discovery rate correction*.

#### Regional brain analyses

**Figure 7:**
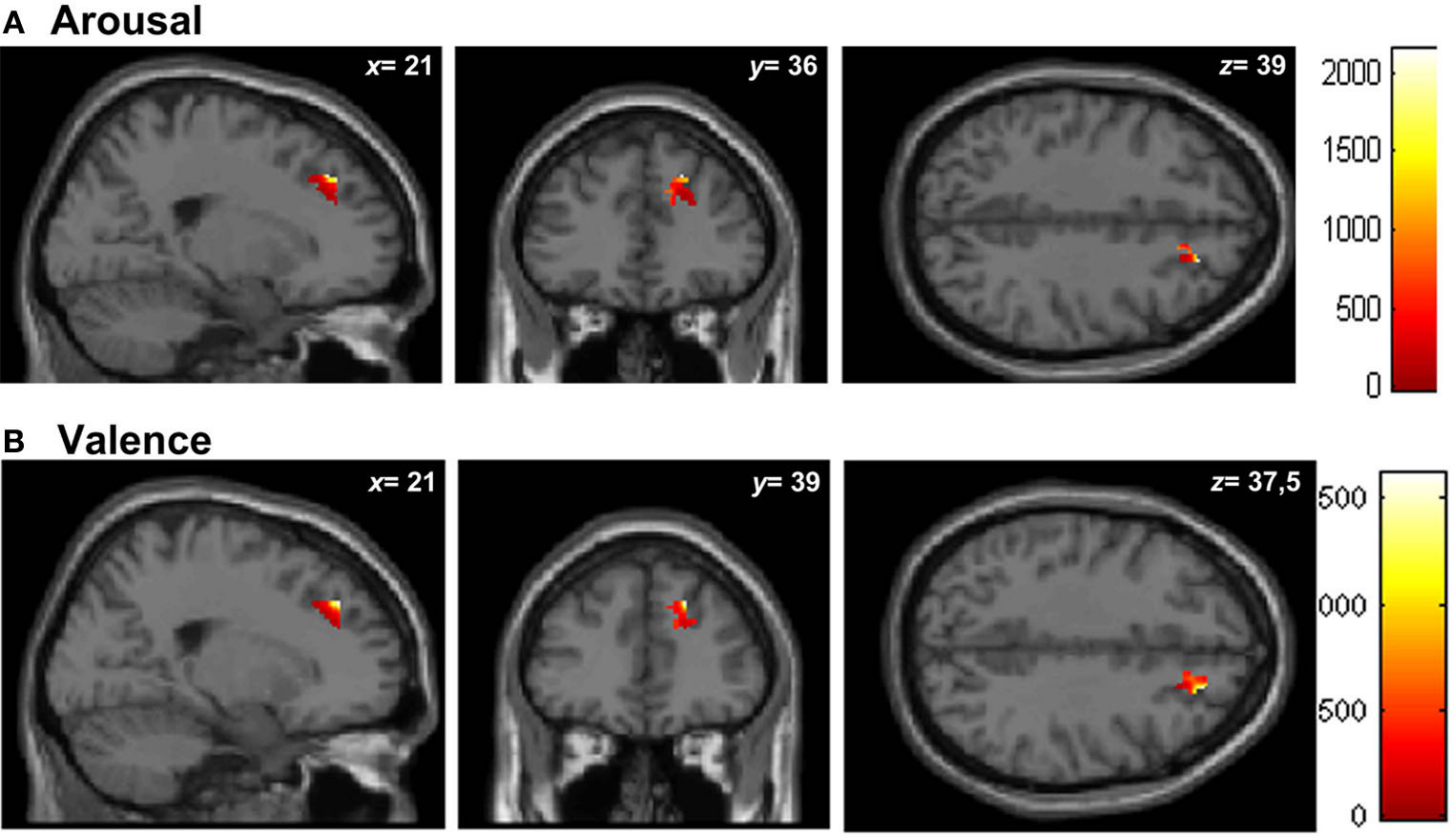
**Graphic display of regional gray matter patterns of intensity using the lesion overlap results of the VLSM analysis (BA9/32) correlated with task performance in controls. (A)** Arousal and **(B)** Valence rating differences.

Similar results were found in the regressions done at the patient's lesion ROI. HC showed greater gray matter amount in the right SFG (BA 9-32) for higher arousal-related REAPPself ability scores (LNeg-Dec; see Figure 7A), as well as for the valence domain (see Figure 7B). The MNI coordinates are presented in Table 6.

In summary, following results were obtained: First, we found an association between deficits in REAPPself ability performance and a dlSFG area overlapping the right SFG (BA9/32) using exploratory VLSM analysis. Second, repeated-measures ANOVA confirmed that the ROI group was less successful in regulating negative emotional responding (i.e., poorer REAPPself ability) compared to IntactROI and HC groups. Third, regional VBM analyses of the mentioned areas in the HC revealed that REAPPself ability was positively related to BA9/32 gray matter intensities.

## Discussion

The cognitive regulation of negative emotions is crucial for mental health, yet there is a lack of lesion studies investigating reappraisal. Therefore, the goal of this study was to identify critical PFC regions for reappraisal ability by analyzing the performance of brain-damaged patients and healthy participants. Taking in consideration the cognitive difficulties of the patients, we thoroughly trained REAPPself assuring that the participants apply the proper strategy. VLSM analysis among the patients and VBM analysis of the healthy control's gray matter showed that specific regions of the right SFG including the dlPFC (BA9) and the dACC (BA32) might be indispensable for REAPPself. Furthermore, ROI-based group comparisons supported the results, demonstrating that a lesion located in the mentioned areas significantly impaired down-regulation of negative arousal. To the best of our knowledge, the current study is the first lesion study using neuroimaging methods for the identification of circumscribed brain regions indispensable for the REAPPself ability.

### The SFG and reappraisal of negative stimuli

In line with previous investigations, the current study linked down-regulation of negative emotions by REAPPself to regions near the mPFC (Ochsner et al., 2004). Our results were also in accordance with latest fMRI data, as six from the 7 fMRI studies investigating down-regulation of negative emotions by REAPPself (as classified by Ochsner et al., 2012) have consistently demonstrated the involvement of areas in the depicted SFG, including the dlPFC (Erk et al., 2010), dorsomedial PFC (Schardt et al., 2010), and dACC (Koenigsberg et al., 2010; Lang et al., 2012). Given that the first descriptive comparison between HC and the whole patient group showed no significant differences, the current ROI groupwise comparisons demonstrate that a lesion in the target region was indeed crucial for REAPPself, especially for the down-regulation of arousal. Interestingly, a previous study has discussed that REAPPself might be particulary effective in the down-regulation of physiological responding (Shiota and Levenson, 2012).

Current results can be interpreted in the frame of a cognitive control of emotions based on anatomical connections between (1) ACC, insula and basal ganglia (Ongur and Price, 2000; Ibanez et al., 2010; Ibanez and Manes, 2012), (2) amygdala-OFC-ACC (Carmichael and Price, 1995; Cavada et al., 2000) as well as (3) dlPFC and basal ganglia (Heekeren et al., 2008). The SFG (BA6/BA8/BA9/BA32) can be divided in an anteromedial (amSFG), a dorsolateral (dlPFC) and a posterior region (pSFG), and is also supposed to be involved in several cognitive control tasks (du Boisgueheneuc et al., 2006; Moreno-Lopez et al., 2012; Li et al., 2013). Moreover, the SFG is highly interconnected, with pathways extending to the ACC, middle frontal gyrus, inferior frontal gyrus (IFG) as well as thalamus and caudate nucleus in the basal ganglia. This important PFC region lies in a unique position between emotional limbic regions and highly cognitive and executive process networks in the dorsal and medial areas of the PFC (Li et al., 2013). The dACC is also one of the few PFC areas that presents strong projections to amygdala nuclei, and might be the cue connection between prefrontal executive and limbic emotional areas during ER (Ray and Zald, 2012). Furthermore, our findings support previous studies in showing that the activation of dACC regions predicts cognitive reappraisal success (Ochsner et al., 2002). This assumption has been supported by *neurofeedback* techniques, in which the down-regulation of emotion-related insula activity was accompanied by the right SFG including ACC (BA32) involvement during reappraisal of threat-related stimuli (Veit et al., 2012). Moreover, conscious self-regulation of brain activity (e.g., right SFG top down control) may depend on an interaction with unconscious subcortical processes, involving not only emotional (amygdala) but also motor skill learning (basal ganglia) as shown in recent models of neurofeedback (Birbaumer et al., 2013).

### Right SFG lesion and REAPPself impairments

Several studies have demonstrated that the ROI depicted by our VLSM results (including the right dACC) is relevant not only for REAPPself, but also for inhibitory control, an executive function that excludes irrelevant information from WM in order to prevent undesirable behavioral responses (Garavan et al., 1999; Vanderhasselt et al., 2012). Thus, REAPPself of negative stimuli may imply the inhibition of dominant negative thoughts, permitting a detached third-person perspective. For instance, a recent study showed that a habitual reappraisal use is positively associated with the ability to inhibit dominant thoughts to negative cues (Vanderhasselt et al., 2013). Similarly, Salas et al. (2014) present a reappraisal model, in which behavioral inhibition is presented as an essential skill for reappraisal generation (Salas et al., 2014). Accordingly, our results showed that inhibition failures during the Go/NoGo task, assessed by the number of errors, were positively correlated with the raw scores of arousal in the Dec condition. Moreover, the ROI group not only showed deficits in REAPPself, but was also the group with most inhibition failures (number of errors) during Go/NoGo tasks. This is of particular interest, as one case study reported that inhibition impairments after a left frontoparietal lesion generated difficulties to spontaneously generate reappraisals (Salas et al., 2013). As previously shown in lesion studies, patients with right PFC lesions typically show inhibition difficulties, reflected by increased error rates in the interference condition of the Stroop task (Vendrell et al., 1995). Furthermore, studies investigating lesions of the right ACC and dlPFC regions report inhibition deficits (Turken and Swick, 1999; Swick and Turken, 2002), rule breaking and difficulties in strategy planning (Burgess et al., 2000). Considering this findings, it would be expectable to spot more right lateralized regions comprising overlapping areas for cognitive inhibition as being crucial for REAPPself (i.e., detaching) of negative events, which in turn, implies the inhibition of negative meanings. However, as inhibition failures (number of Go/NoGo errors) did not significantly correlate with the REAPPself ability scores (LNeg-Dec), our findings support the reappraisal model of Salas et al. (2014). That is, inhibition failures might have influenced reappraisal generation (Salas et al., 2014). As Go/NoGo errors were positively associated with the amount of negative arousal in the Dec condition (i.e., the more errors, the more arousal), it might be interpreted that automatic negative meaning of the stimuli could not be inhibited appropriately. For instance, our results support the findings of McRae et al. (2012a,b), who did not find significant associations of general reappraisal ability with response inhibition, but with WM capacity (McRae et al., 2012b). Therefore, our results revealed that REAPPself might not be dependent only on dACC and inhibition, but also on WM process and strategy planning functions that rely on dlPFC areas (Heyder et al., 2004; Kaller et al., 2011, 2013).

A previous fMRI study examining the contribution of PFC areas in ER shows that the right dlPFC is strongly involved in reappraisal function, regardless of the kind of stimuli that are reappraised (Golkar et al., 2012). This is not surprising, as reappraisal relies on executive functions that update emotional to new non-emotional “reappraised” thoughts and maintain these reinterpretations in mind (Malooly et al., 2013). The displayed areas in the dlSFG region enclose the dlPFC areas in BA9, which have not only been linked to WM and executive processes, but also with metacognitive evaluations of oneself and others, particularly the right dlPFC (Schmitz et al., 2004). That is, the right PFC might be recruited when self-evaluations are produced. For REAPPself, a self-focused strategy, evaluations about the self might be essential. Moreover, since a part of the identified ROI is placed in the white matter of the PFC (between BA9 and BA32), REAPPself ability might also be dependent on the interaction and connectivity of the mentioned areas. Further analysis of SFG connectivity and the influence on REAPPself might clarify these issues.

Interestingly, the ROI group showed not only more inhibition deficits, but also more immediate memory recall and processing speed deficits, as well as lower fluid intelligence scores as the IntactROI and HC group. Therefore, a lesion in the depicted region might lead to other cognitive impairments, besides of those of REAPPself and inhibition. These variables showed no significant correlations with REAPPself ability scores, but with the raw arousal and valence scores of the Dec condition. In other words, these cognitive variables had a significant influence on the amount of negative emotion during reappraisal. Here, results showed that cognitive abilities such as fluid intelligence and immediate memory, correlated negatively with arousal and valence scores during the Dec condition. This result might support the assumption of less negative affect through heightened cognitive control abilities (Williams et al., 2009).

### ROI lesion and depression symptoms

The current findings show that the ROI group suffered more from depression than the two other groups. These results are in agreement with previous studies investigating lesions in the right hemisphere, which also show associations with impaired affective processing, reflected by the presence of anxiety and depression (Berg et al., 2000; Zorzon et al., 2001, 2002). In addition, our results support the findings of Königs and colleagues, which demonstrated increased vulnerability for depression after a bilateral dlPFC lesion (Koenigs et al., 2008). Apart from this, depression was also positively associated with the amount of negative emotionality during the Dec condition. Depression and other affective disorders have been related to inhibition failures of negative stimuli (Joormann and Gotlib, 2008; Joormann, 2010) and to impaired reappraisal (Johnstone et al., 2007; Moore et al., 2008; Ehring et al., 2010). For these reasons, depression might reflect a confounder; particularly for the analysis of valence rating scores (were depression was a significant influence). This finding might lead to the assumption that the depicted right SFG region might be important for a valence-related REAPPself ability, but probably not indispensable, as it might be with an arousal-related REAPPself ability. In addition to the lesion, depression might have influenced the subjective down-regulation of negative valence.

### VBM results of gray matter in healthy controls

Our whole-brain VBM findings showed that partially different neural structures were correlated with arousal- and valence-related REAPPself ability. For arousal REAPPself, we found positive associations with more subcortical regions as the insula, whereas valence was associated with highly cognitive areas as the middle and inferior frontal gyrus, as well as with the inferior parietal lobule. Although both of the constructs are assumed to be difficult to separate in the subjective experience (Kuppens et al., 2013), the obtained results lead to the assumption that arousal down-regulation comprise the involvement of limbic regions mainly related to emotional awareness and physiological responding, whereas valence down-regulation is a more elaborated process, in which highly cognitive regions are involved (Citron et al., 2014). Accordingly, insula activity has been consistently observed during changes in autonomic arousal (Critchley et al., 2002, 2003). However, in studies examining the evaluation of valence, more cortical, attentional structures are observed (Kensinger, 2004; Kensinger and Corkin, 2004). In addition, the SFG was significantly associated with the down-regulation of both, arousal, and valence self-reports. This was confirmed by the regional VBM analysis, were right SFG regions showed significant positive correlations with REAPPself ability. These findings support previous evidence showing that the anatomical volume of ACC (BA32) is positively associated with a cognitive reappraisal ability (Giuliani et al., 2011).

### Implications for further research

Summarizing, the involvement of SFG regions during reappraisal of negative stimuli has been strongly underlined in the majority of fMRI studies, and included in several reappraisal models (Ochsner et al., 2002, 2004, 2012; Wager et al., 2008; Koenigsberg et al., 2010; Buhle et al., 2013). Inhibition performance, which is supposed to be a right lateralized function (Garavan et al., 1999), might influence the ability of decreasing automatic negative appraisals, thus constituting a corner pillar for the architecture of reappraisal and especially, REAPPself (Salas et al., 2013, 2014). However, we cannot rule out the influences of depression symptoms, particularly on the down-regulation of valence. Although inhibition, depression and impaired REAPPself ability are strongly associated (Joormann and Gotlib, 2008; Joormann, 2010; Aldao and Nolen-Hoeksema, 2012; Barnow et al., 2013), additional research is needed to explain the direction of these associations with a bigger sample. Analyses about these associations are, unfortunately, outside the scope of this work. Nevertheless, to gain further insight into the effects of a trained modulation of the right SFG (specifically dACC and dlPFC areas) on REAPPself performance and their related limbic responses might be useful in the treatment of various psychiatric disorders involving emotional dysregulation. Furthermore, it would be of great interest to examine whether teaching patients to gain control over the neural activity (right SFG and related subcortical networks) via neurofeedback would yield positive therapeutic effects.

### Limitations

No study is free of limitations or possible improvements. In the present study, the sample size was not large enough to reach significance with conventional multiple-measure corrections (see for example Medina et al., 2010). Therefore, our VLSM results have to be interpreted with caution due to the risk of false-positive findings. Additional research should replicate these findings with a larger sample of patients. However, the ROI based group comparison supported the VLSM results. Furthermore, although we were very conservative in lesion reconstruction, we cannot rule out the possibility of etiology and treatment confounders. We therefore controlled for the lesion volume in the statistical analysis, and it was not significantly different between groups. However, we have to take the influence of cortical reorganization of functions by slow-growing tumors into consideration (Desmurget et al., 2007). It is also important to mention that the infiltration pattern of brain tumors is diffuse *per se* and generally difficult to assess. The growth of such a tumor results in T2-weighted hyperintense signal alteration; the current methods in MRI make it impossible to differentiate between tumor and perifocal edema, as both features may lead to the MRI pattern (Essig et al., 1998). However, the employed T2-FLAIR sequence is regarded to be one of the most sensitive MRI sequences to detect the extensiveness of damaged brain tissue. Thus, by considering the whole T2 hyperintensity, the analysis was performed conservatively, as this type of segmentation includes the maximum area of damaged brain tissue, detectable with current methods. The highly educated control group might also represent a source of bias, although this variable did not significantly correlate with any of the outcome variables and no significant differences in demographic variables were found in the ROI group-wise comparison. Finally, although previous investigations studying reappraisal function in brain-damaged patients argue that reappraisal ability might be a problematic variable to measure due to the potential cognitive impairments of brain-damaged patients (Salas et al., 2014), our results show that these reappraisal difficulties might be dependent of the localization of the lesion (right SFG), as the descriptive patients-HC group comparison did not show any significant results in reappraisal ability (except for the significant influence of depression).

## Conclusions

Considering our limitations, it is safe to conclude that the integrity of the right dACC and dlPFC might be of crucial importance not only for REAPPself ability, but also for affective and cognitive health. To the best of our knowledge, the current work is the first lesion study on cognitive reappraisal that targets circumscribed brain regions using imaging methods. It brings useful insights in the importance of specific right SFG areas for REAPPself. These findings might have important implications for studies using real-time fMRI techniques (Decharms et al., 2004). It would be of great interest to investigate whether the conscious modulation of right SFG BOLD activity could influence limbic responses using neurofeedback methods. The development of evidence-based neurofeedback trainings would be of prime importance in patients suffering from emotional dysregulation, depression, and other types of psychopathology.

### Conflict of interest statement

The authors declare that the research was conducted in the absence of any commercial or financial relationships that could be construed as a potential conflict of interest.
