# Corrigendum: Detaching from the negative by reappraisal: the role of right superior frontal gyrus (BA9/32)

**DOI:** 10.3389/fnbeh.2014.00264

**Published:** 2014-08-22

**Authors:** Rosalux Falquez, Blas Couto, Agustin Ibanez, Martin T. Freitag, Moritz Berger, Elisabeth A. Arens, Simone Lang, Sven Barnow

**Affiliations:** ^1^Department of Clinical Psychology and Psychotherapy, Institute of Psychology University of HeidelbergHeidelberg, Germany; ^2^Laboratory of Experimental Psychology and Neuroscience (LPEN), Institute of Cognitive Neurology (INECO), Favaloro UniversityBuenos Aires, Argentina; ^3^Diego Portales University, UDP-INECO Foundation Core on Neuroscience (UIFCoN)Santiago, Chile; ^4^Departamento de Psicología, Universidad Autónoma del CaribeBarranquilla, Colombia; ^5^Department of Radiology, German Cancer Research Center (DKFZ)Heidelberg, Germany

**Keywords:** right SFG, reappraisal, VBM, VLSM, lesion

We noticed an error in one of our presented analyses. One mismatched brain image was accidentally included in the voxel-based-morphometry (VBM) analysis. Thus, arousal and valence values were consecutively not properly assigned to the morphological brain data of the other included participants. Of three analyses implemented in this study (VSLM, ROI-based), only the VBM analysis was affected but the others remain untouched. Therefore, we re-conducted the whole VBM analysis with the correct allocation of data. The corrected results showed changes in the whole-brain and regional correlations compared to the originally presented results. However, the correlations in the expected areas of the original manuscript remain significant for arousal and valence difference scores.

Fortunately, these results do not impact the main implications and neither invalidate the conclusions derived from the study nor introduce differing directions of inference. The right superior frontal gyrus (SFG/BA9) and anterior cingulate cortex (ACC/BA32) remain significant at whole-brain *p* < 0.001 uncorrected level, and the ROI analysis still showed significant correlations with gray matter intensities in the right SFG (BA9).

The corrected results affect Figures 6, 7, Tables 5, 6, and small parts in results and discussion which are attached below.

**Figure 6:**
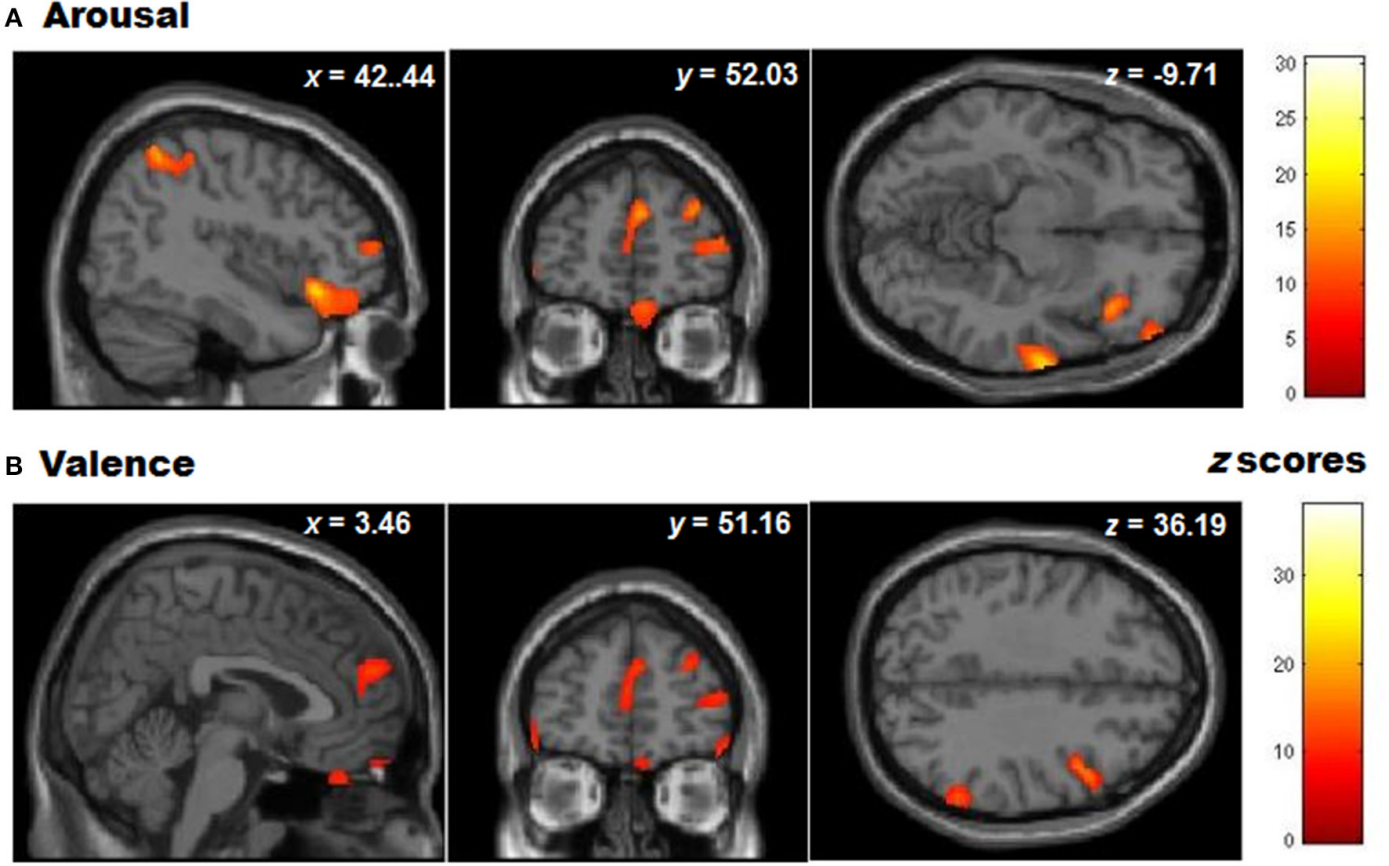
**Whole brain patterns of gray matter volumes correlated with task performance in controls. (A)** Arousal rating differences and **(B)** Valence rating differences (presented at a level of *p* < 0.001 unc).

**Figure 7:**
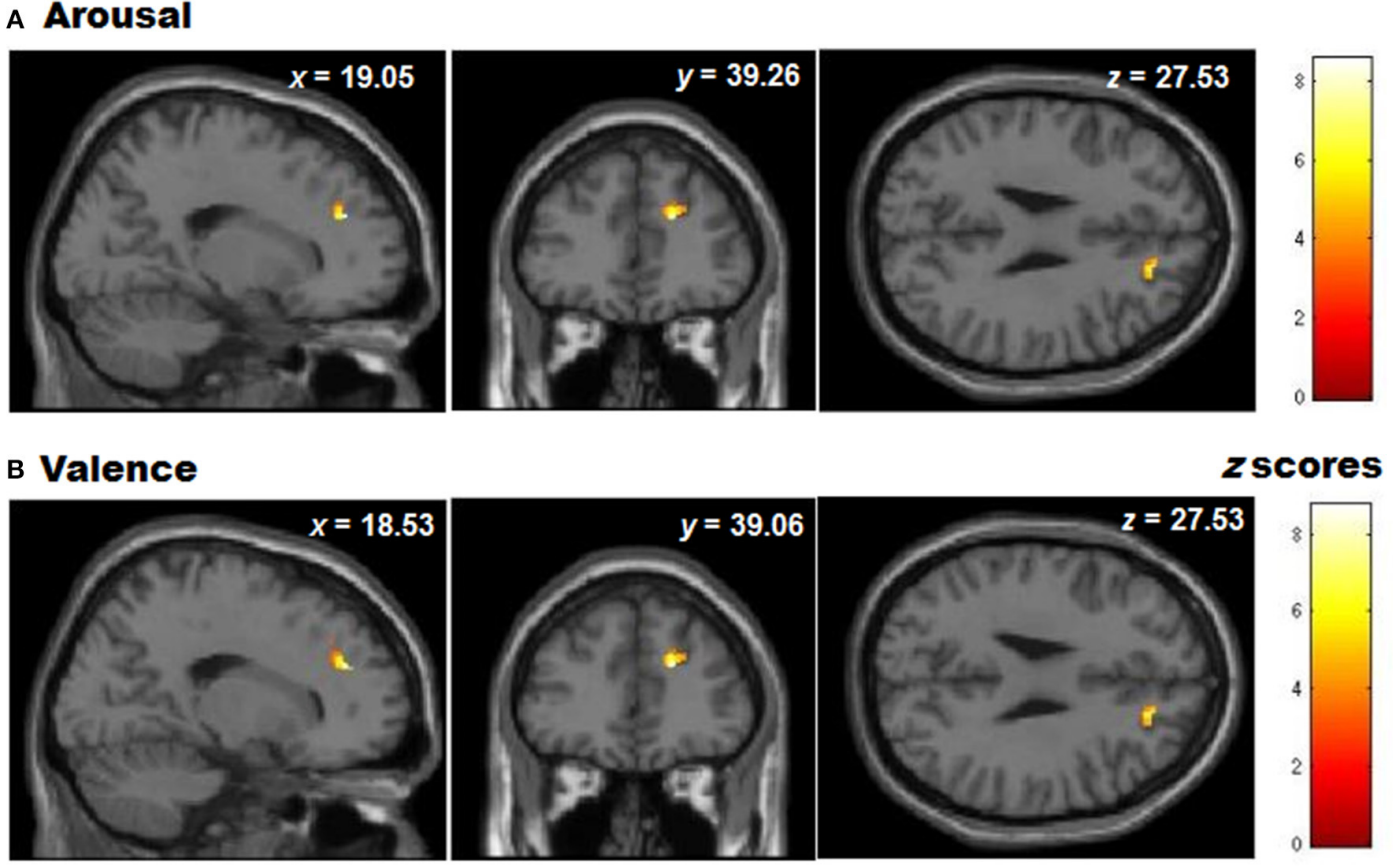
**Graphic display of regional gray matter patterns of volume using the ROI depicted by the VLSM analysis (BA9/32) correlated with task performance in controls for (A) Arousal and (B) Valence rating differences (presented at a level of *p* < 0.05 unc)**.

**Table 5:** **Whole brain Patterns of GM volume correlated with task performance in controls**.

**Region**	**BA**	**Coordinates**			***F* peak**	***p* (unc)**
		***x***	***y***	***z***		
**(A) AROUSAL**						
Superior Temporal Gyrus R	21	67.5	−1.5	−13.5	30.50	<0.001
Inferior Parietal Gyrus R	2	57	−28.5	55.5	23.07	<0.001
Angular Gyrus R	40	58.5	−51	30	20.57	<0.001
Rolandic Operculum L	13	−47	−4	13	20.11	<0.001
Inferior Frontal Gyrus R	47	41	27	−12	18.73	<0.001
		54	43.5	−13.5	16.12	<0.001
Mid Occipital Gyrus L	39	−52.5	−73.5	13.5	18.65	<0.001
Orbital Gyrus R	11	7.5	52.5	−22.5	11.83	<0.001
Inferior Parietal Gyrus L	40	−55.5	−34.5	48	15.71	<0.001
Superior Parietal Gyrus L	40	−37.5	−64.5	58.5	14.83	<0.001
Superior Frontal Gyrus R	9	6	52.5	25.5	15.33	<0.001
Anterior Cingulate R	32	1.5	48	13.5	14.82	<0.001
Mid Frontal Gyrus R	10	33	55.5	27	14.86	<0.001
	46	45	54	9	11.64	<0.001
Mid Frontal Gyrus L	8	−25.5	33	46.5	14.04	<0.001
Superior Frontal Gyrus R	10	36	55.5	9	10.96	<0.001
Parahippocampal Gyrus R	34	15	−9	−22.5	10.56	<0.001
Superior Frontal Gyrus L	10	−3	63	10.5	10.58	<0.001
**(B) VALENCE**						
Mid Temporal Gyrus R	21	64.5	−1.5	−15	37.87	<0.001
Parahippocampal Gyrus R	28	22.5	−9	−27	22.70	<0.001
Inferior Parietal Gyrus R	2	57	−28.5	55.5	22.26	<0.001
Angular Gyrus R	40	60	−52.5	28.5	17.18	<0.001
Mid Temporal Gyrus R	39	54	−60	21	13.27	<0.001
Inferior Frontal Operculum R	9	43.5	9	36	16.42	<0.001
Inferior Frontal Gyrus R	47	42	28.5	−13.5	10.38	<0.001
Parahippocampal Gyrus L	–	−24	−1.5	−30	15.41	<0.001
Superior Parietal Gyrus R	40	40.5	−55.5	58.5	15.12	<0.001
Mid Frontal Gyrus R	10	33	55.5	27	14.84	<0.001
Superior Frontal Gyrus R	11	7.5	54	−22.5	10.16	<0.001
Rolandic Operculum L	13	−46.5	−3	12	13.91	<0.001
Superior Frontal Gyrus R	9	4.5	54	24	12.87	<0.001
Anterior Cingulate L	32	0	49.5	13.5	12.26	<0.001
Mid Frontal Gyrus L	8	−25.5	33	46.5	12.72	<0.001
Postcentral Gyrus L	40	−54	−34.5	54	12.63	<0.001
Mid Frontal Gyrus R	46	46.5	54	10.5	12.02	<0.001
Lingual Gyrus L	30	−16.5	−40.5	−9	11.29	<0.001
Superior Temporal L	20	−45	−9	−16.5	11.30	<0.001
Superior Frontal L	10	−3	63	10.5	10.59	<0.001

VBM, voxel-based morphometry; BA, Brodmann area; R, right; L, left; GM, gray matter.

**Table 6:** **Regional brain Patterns of GM volume correlated with task performance in controls**.

**Region**	**BA**	**Coordinates**			***F* peak**	***p* (FWE-cor)**
		***x***	***y***	***z***		
**(A) AROUSAL**						
Superior Frontal Gyrus R	9	19.5	40.5	25.5	8.57	0.037
**(B) VALENCE**						
Superior Frontal Gyrus R	9	19.5	40.5	25.5	8.74	0.035

VBM, voxel-based morphometry; BA, Brodmann area; R, right; L, left; GM, gray matter; FWE, family-wise error correction.

The authors deeply regret this error and apologize for any confusion it might have caused.

## Results

The sentence in the results section for the arousal scores

“… more specifically the right SFG (BA 9–32; see Figure 6A), left insula, basal ganglia and mid temporal gyrus, and bilateral cerebellum (See Table 5A for MNI coordinates).”

should be replaced by:

“… more specifically the right SFG (BA 9–32, see Figure 6A), orbital gyrus, leftmiddle occipital gyrus, right angular gyrus, superior temporal gyrus, left rolandic operculum, right inferior parietal cortex and bilateral mid-frontal gyrus (See Table 5A for MNI coordinates).”

The sentence in the results section for the valence scores

“For the valence domain, this relation appeared at the left SFG (BA 9–32; see Figure 6B), right SFG, left mid and inferior frontal gyri, temporal cortex, parietal cortex, basal ganglia and cerebellum (See Table 6B, for MNI coordinates).”

should be replaced by:

“For the valence domain, this relation appeared at the left SFG (BA10; see Figure 6B), right SFG (BA9) including ACC (BA32), left mid and inferior frontal gyri, temporal cortex, parietal cortex, parahippocampal gyri and opercula (See Table 6B, for MNI coordinates).”

## Discussion

The sentences in the discussion section:

“For arousal REAPPself, we found positive associations with more subcortical regions as the insula, whereas valence was associated with highly cognitive areas as the middle and inferior frontal gyrus, as well as with the inferior parietal lobule. Although both of the constructs are assumed to be difficult to separate in the subjective experience (Kuppens et al., 2013), the obtained results lead to the assumption that arousal down-regulation comprise the involvement of limbic regions mainly related to emotional awareness and physiological responding, whereas valence down-regulation is a more elaborated process, in which highly cognitive regions are involved (Citron et al., 2014).”

should be replaced by:

“For arousal REAPPself, we found positive associations with the right orbital gyrus and left middle occipital gyrus whereas the down-regulation of both (valence and arousal) were associated with highly cognitive areas as the middle and inferior frontal gyrus, as well as with the inferior parietal lobule. Although both of the constructs are assumed to be difficult to separate in the subjective experience (Kuppens et al., 2013), the obtained results lead to the assumption that arousal down-regulation comprise the involvement of limbic regions mainly related to emotional awareness and multisensory integration (Ongur and Price, 2000). In contrast, the down-regulation of both arousal and valence is an elaborated process, in which highly cognitive regions are involved. These findings are in accordance with an earlier report, in which arousal and valence are described as separable dimensions (Citron et al., 2014).”

### Conflict of interest statement

The authors declare that the research was conducted in the absence of any commercial or financial relationships that could be construed as a potential conflict of interest.
