# Supplementary material for: Detaching from the negative by reappraisal: the role of right superior frontal gyrus (BA9/32)
Source: Front Behav Neurosci. 2014 May 9;8:165. doi: 10.3389/fnbeh.2014.00165 (PMC4023069; doi:10.3389/fnbeh.2014.00165)
Supplement: Supplementary file 1 [file Presentation1.PDF]

## Supplementary methods

*Cognitive control assessment*

In order to obtain a general profile of the cognitive functioning of all participants, several cognitive screening instruments were used.

1. *Fluid intelligence* was assessed using the German adaptation of the *Culture Fair Intelligence Test- Scale 2* (Cattell, 1960) for the diagnosis of general mental capacity. The paper-and-pencil test comprised four types of figural subtests (series, classifications, matrices, topologies). Answers are given in multiple choice formats, and every subtest has a time limit. In the first subtest (series), participants have to complete series of figures by choosing one out of five alternatives as the correct continuation. The subtest "classifications" required the participants to select which out of five figures did not correctly match the others. In the third subtest (matrices), participants were instructed to identify which figure out of five completed a set of three pictures. In the last subtest (topologies), participants have to identify the figure which was created according to the same principle as a sample stimulus (logical reasoning test).
2. *Processing speed and memory performance* were measured using the nonverbal cognitive screening of the Aphasia-Check-List (verbal fluency task and nonverbal memory task from the ACL; Kalbe et al., 2002;Kalbe et al., 2005).
    a. The *verbal fluency* task was divided in two parts, a phonemic and a categorical fluency part. In the phonemic verbal fluency part, participants were instructed to retrieve as many words as possible in accordance to the rule "word starts with letter B", in a limited amount of time (1 min.). Then, in the categorical verbal fluency part, participants had to retrieve words in the same amount of time after another rule, namely "word belongs to the category supermarket".
    b. *Memory performance* was assessed using a recognition paradigm with six geometric figures. Patients were asked to memorize the figures within ten seconds. Following, patients had to recognize the six correct figures from a set of 15 figures in an immediate (immediately after memorizing) and a delay recall (10-minute delay). Thereby, nonverbal short-term and intermediate-term memory

performance is calculated by subtracting the number of errors from the number of correct recognized figures.

3. *Cognitive flexibility* assessment was administered using the *Trail Making Task A, B* (TMT; Tombaugh, 2004). This paper-pencil task was divided into two subtasks (Part A and B). In part A, participants had to draw a line connecting 26 encircled numbers distributed on a sheet of paper in the right order as fast as they could. Part B presented not only encircled numbers, but also letters on a sheet of paper. Here, participants had to alternate between numbers and letters in the right order (e.g., 1, A, 2, B, 3…). Task performance time was noted in both subtasks. Both tasks had a practice sheet, where participants were allowed to make errors and practice till they understood the rules. The whole task duration was ca. 3 minutes. Cognitive flexibility scores were calculated by subtracting the time required in Part A from the time required in Part B.
4. *Behavioral Inhibition* was measured using the computerized *Go/NoGo task* of the German *Test for Attentional Performance* (TAP 2.3, Psytest; Zimmermann and Fimm, 2002), a valid and reliable test battery (Jakobsen et al., 2011). The task required participants to respond to a Go stimulus, an x, by pressing a button, while inhibiting the response to a visually similar NoGo stimulus, a + sign. Task completion took 15 min, including practice trials. Number of Errors (NoE) and reaction times (RT) are recorded.

*Affective screening*

The assessment of emotional functioning was conducted using the Beck’s Depression Inventory (BDI-II; Kuhner et al., 2007). The BDI-II is a 21-item self-report measure of depression severity applied in various clinical and nonclinical settings. Participants were instructed to rate every item honestly on a 4-point scale (0-3). Summary scores range between 0 and 63.
